# Translation, cross-cultural adaptation and validation of the Chinese version of irritable bowel syndrome severity scoring system

**DOI:** 10.3389/fmed.2026.1779402

**Published:** 2026-05-07

**Authors:** Qian Li, Jia-Hao Mo, Hui-Qi Chen, Meng-Xin Chen, Xiao-Jia Ni, Ze-Hui He, Bing-Xiong Ou, Xiu-Cai Fang, Xiao-Bo Yang

**Affiliations:** 1 State Key Laboratory of Dampness Syndrome of Chinese Medicine, The Second Affiliated Hospital of Guangzhou University of Chinese Medicine, Guangzhou, Guangdong, China; 2 Chinese Medicine Syndrome Research Team, The Second Affiliated Hospital of Guangzhou University of Chinese Medicine, Guangzhou, Guangdong, China; 3 The Second Clinical College of Guangzhou University of Chinese Medicine, Guangzhou, Guangdong, China; 4 Guangdong Provincial Key Laboratory of Research on Emergency in Traditional Chinese Medicine, Guangzhou, Guangdong, China; 5 Big Data Research Center of Chinese Medicine, The Second Affiliated Hospital of Guangzhou University of Chinese Medicine, Guangzhou, Guangdong, China; 6 Department of Pharmacy, The Second Affiliated Hospital of Guangzhou University of Chinese Medicine, Guangzhou, Guangdong, China; 7 Departement of Gastroenterology, Peking Union Medical College Hospital, Chinese Academy of Medical Sciences and Peking Union Medical College, Beijing, China

**Keywords:** Chinese version, irritable bowel syndrome, irritable bowel syndrome severity scoring system, translation and cultural adaptation, validation

## Abstract

**Objective:**

Given that the Irritable Bowel Syndrome Severity Scoring System (IBS-SSS) is widely utilized in IBS-related clinical research but lacks an officially approved Chinese version (IBS-SSS-C), which has impeded its application in Chinese research contexts. This study aims to translate and cross-culturally adapt the IBS-SSS into a Chinese version, and validate its key properties, thereby developing a reliable and officially recognized tool for assessing IBS severity among the Chinese population.

**Methods:**

We obtained translation permission for the IBS-SSS from the Rome Foundation, and translated and cross-culturally adapted it into a Chinese version according to the official guideline. Validation was performed as clinical trials assessing responsiveness to change (*n* = 95), test–retest reliability (*n* = 35) and the floor-ceiling effects (*n* = 95) of IBS-SSS-C. We assessed the IBS-SSS-C score’s responsiveness to change using adequate relief (AR) as an anchor.

**Results:**

Through forward and backward translation, and cross-cultural adaption, the IBS-SSS-C was developed and approved for use by the Rome Foundation. A significance difference in the absolute change of the IBS-SSS-C total score before and after treatment was found in the AR responder and non-responder groups (*p* = 0.001). This trend also emerged for the items abdominal pain severity (*p* = 0.032), abdominal pain frequency (*p* = 0.001), and bowel habit satisfaction (*p* = 0.036). There was good test–retest reliability (*r* = 0.72) for the IBS-SSS-C total score, and a moderate to good correlation (*r* = 0.41–0.81) for each of its items. No floor or ceiling effect was found in either IBS-SSS-C or any of its item.

**Conclusion:**

Our findings indicate that the IBS-SSS-C appears to be a reliable and appropriate instrument for IBS outcome assessment in the Chinese population; nevertheless, further validation in larger-scale, multi-center studies is required.

## 1 Background

Irritable Bowel Syndrome (IBS) is one of the most studied functional gastrointestinal disorders (1). It is characterized by chronic recurring abdominal pain with abnormal stool appearance and/or frequency (2). Epidemiological studies have shown that IBS affects between 2 and 6% of the population in most geographical regions (3), and between 4.6 and 6.5% of Chinese population (4, 5). IBS is associated with significant reductions in both health-related quality of life and social functioning. It also imposes a heavy economic burden on health care systems. For example, estimates of annual healthcare costs exceed US $10 billion in the United States (6), €8 billion in Europe (7), and ¥123 billion in China (8).

Because of the absence of a physiological marker specific to IBS, outcome measures in clinical trials rely on patients’ self-reported symptoms, and draw support from assessment tools. However, at present only a few standardized assessment tools for IBS are available. The Irritable Bowel Syndrome Severity Scoring System (IBS-SSS) is one of the few standardized tools available for IBS outcome assessment with confirmed validation (9). This scale has been widely accepted and used for clinical studies in many countries for nearly 25 years (10–12). The IBS-SSS has been shown to have several advantages over other available IBS outcome measure tools, as it can produce a more detailed assessment of IBS symptoms and severity (13). The IBS-SSS not only focuses on common IBS symptoms, including abdominal pain, abdominal distension and bowel habit, but also patients’ quality of life. Therefore, it is also used as a global assessment tool for IBS (14–16).

The IBS-SSS was established by Whorwell et al. (9) and written in English. The original version’s sensitivity to change and reproducibility have been confirmed by the authors (9). The IBS-SSS has been translated into many other languages, such as German (17), Spanish (18) and Japanese (19), and it is widely used in IBS-related clinical studies. However, there is currently no Chinese version of IBS-SSS with official approval, and this has hindered the application of the IBS-SSS in Chinese research. To address this concern, we have translated, culturally adapted the IBS-SSS and verified the responsiveness to change and test–retest reliability of the Chinese version of the IBS-SSS (IBS-SSS-C) among Chinese populations. Finally, we provide a valid IBS-SSS-C for Chinese researchers.

## 2 Materials and methods

### 2.1 Scale

#### 2.1.1 Translation and cross-cultural adaptation

**Figure 1:**
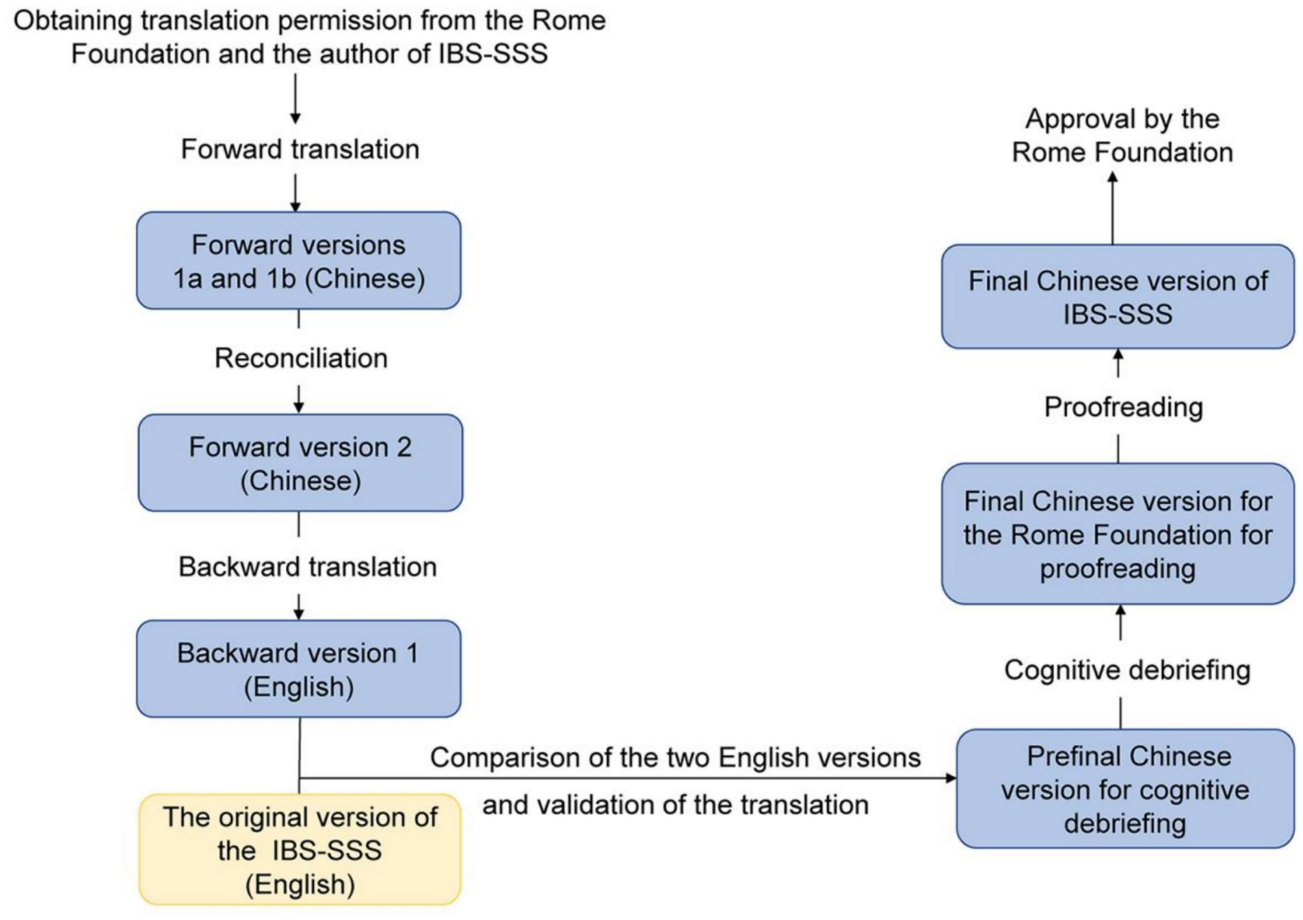
Flowchart of the translation. IBS-SSS: Irritable Bowel Syndrome Severity Scoring System.

We obtained translation permission from the Rome Foundation and the author of the IBS-SSS (Prof. Whorwell). We translated and cross-culturally adapted the English version of IBS-SSS into Chinese according to *The Guidelines for the Translation of Rome Foundation Material*. A flowchart of the translation is shown in the Figure 1.

#### 2.1.2 Validation

After finishing the IBS-SSS-C, we validated it among a sample of the Chinese population. The original version of the IBS-SSS consists of two parts: Part 1 (severity score) and Part 2 (other IBS data). Part 1 is used to record IBS symptom severity scores, while Part 2 is used to record IBS symptoms in details which are not appropriated for scoring. Thus, we only validated Part 1 of the IBS-SSS-C. IBS patients were recruited from the Second Affiliated Hospital of Guangzhou University of Chinese Medicine for the purposes of assessing the IBS-SSS-C’s (1) responsiveness to change, (2) test–retest reliability, and (3) floor and ceiling effects. All patients for validation were from two studies regarding IBS: 35 patients from Study 1 (between November 30, 2017 and February 1, 2020) (20), and 60 patients from Study 2 (between May 11 and December 5, 2018) (21). These studies were approved by the Ethics Committee of Guangdong Provincial Hospital of Chinese Medicine (number: B2016–132-01 and BF2018-031-01), and registered on the website chictr.org.cn (ID: ChiCTR-IOR-17010600, 9 February 2017; ID: ChiCTR1800015641, 12 April 2018). Written informed consent was obtained from each participant. The study protocols used have been well clarified in existing literature (20, 21).

##### 2.1.2.1 Responsiveness to change

**Figure 2:**
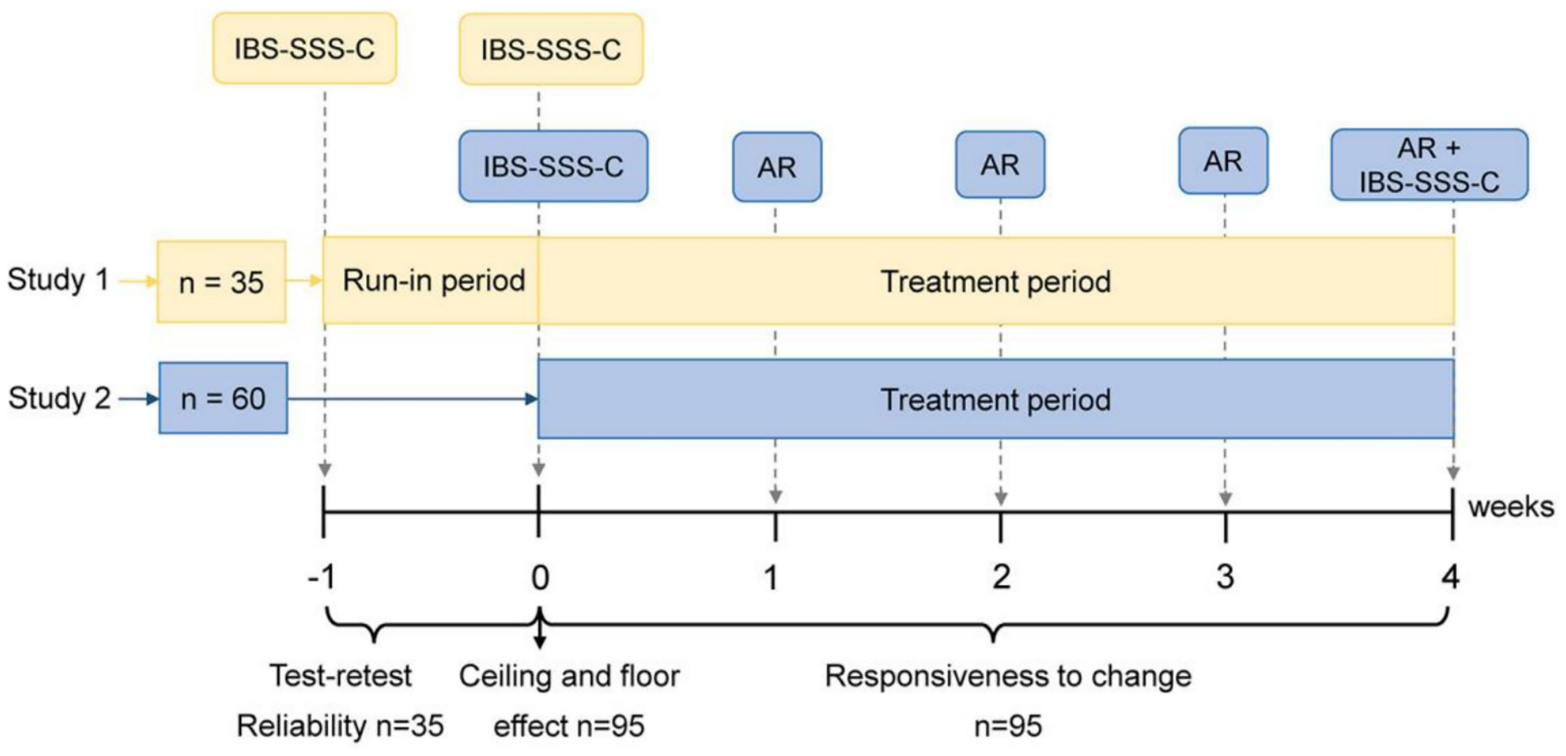
Flowchart of the IBS-SSS-C validation. AR, adequate relief; IBS-SSS-C, Chinese version of Irritable Bowel Syndrome Severity Scoring System.

Ninety-five eligible patients from Study 1 and 2 were included in the analysis of the IBS-SSS-C’s responsiveness to change (Figure 2). All 95 patients took CM decoction or granules twice a day, 30 min after meal times, for 4 weeks. Many studies have confirmed the benefits of Chinese medicine (CM) in IBS treatment (22–25). Details on the CM treatment used in this study are described in existing literature (20, 21). During the treatment period, the patients had to respond weekly to the question “In the past 7 days, have you had adequate relief of your IBS pain and discomfort?” Those patients who answered “yes” for at least 2 weeks during the 4-week treatment period were defined as responders (26, 27). Accordingly, patients were divided into adequate relief (AR) responder and non-responder groups, and we analyzed the absolute change in IBS-SSS-C total score and each item score, before (week 0) and after treatment (week 4) between groups.

##### 2.1.2.2 Test–retest reliability

Thirty-five patients from Study 1 were asked to complete the IBS-SSS-C at week −1, and then asked to return 1 week (±2 days) later (Figure 2). During the interim, patients received no treatment for IBS, and none of them knew that they would repeat the exercise after a week.

##### 2.1.2.3 Floor and ceiling effects

Ninety-five eligible patients from Study 1 and 2 were included in the analysis of the IBS-SSS-C’s floor and ceiling effects (Figure 2). If at week 0, more than 15% of the patients had achieved the highest or lowest possible scores, then we concluded that there was a floor or ceiling effect (28).

#### 2.1.3 Statistical analysis

From the baseline to each time point, categorical variables will be described with frequencies and percentages, and continuous variables will be described with either mean and standard deviation for data with a normal distribution, or median and interquartile range for any non-normally distributed data. IBS-SSS-C total score and each item score were calculated to clarify whether there was a floor or ceiling effect. The absolute change in IBS-SSS-C total score and each item score before and after treatment between AR responder and non-responder groups was analyzed using a student’s t-test or Mann–Whitney test with a two-sided significance level of 0.05. The correlation between the IBS-SSS-C scores at week −1 and week 0 was analyzed using the intraclass correlation coefficient (ICC). All statistical analyses were performed by a professional statistician using IBM SPSS Statistics for Windows, version 26.0 (IBM Corp., Armonk, N. Y., United States) (29).

## 3 Results

### 3.1 Translation and cross-cultural adaptation

We finished the IBS-SSS-C in accordance with the official requirements. Firstly, two translators (Qian Li and Xiaojia Ni) finished the Chinese translation independently, and developed forward versions 1a and 1b, respectively. Both translators have medical backgrounds and experience in medical translation. Neither of them modified, appended, or deleted any of the items. Under the supervision of Xiucai Fang, who was appointed by the Rome Foundation, the two forward versions were checked and any differences in items were identified. The consistency of the two forward versions was satisfactory. Any disagreement was discussed until a consensus was reached. After appropriate modification, version 2 of the forward was developed.

An English-native speaker, who did not participate in forward translation and reconciliation, translated forward version 2 back into English, and developed backward version 1. The translator is a professionally trained Mandarin-English consecutive interpreter and academic English editor with over 7 years’ experience, primarily in healthcare. Under the supervision of Prof. Fang, the original scale and the backward version 1 were compared, item-by-item. The language similarity and interpretation comparability were satisfactory. After that, the pre-final Chinese version was determined.

Five IBS patients were tested to assess the translation in terms of clarity, cultural adaptation, language level, and acceptability. The tests were conducted at the hospital. Participant recruitment was via clinician referrals from the gastroenterology clinics. IBS patients who met Rome IV criteria and agreed to join this survey were required to complete the prefinal Chinese version of IBS-SSS. After finishing the scale, each patient was given a short interview to assess their understanding of each item. The process was conducted by an investigator (Qian Li), who was familiar with interviewing methods. All patients had a good understanding of most items in the translation. Items that some patients were unclear were modified according to their comments and those of Prof. Fang. The final version was based on a consensus among Prof. Fang, translators and the five patients. After submitting all required documents, including a translation report, interview report and language versions for each step, the Rome Foundation approved our IBS-SSS-C (Supplementary material).

### 3.2 Validation

Ninety-five eligible patients were included in this study. The mean age was 36.3 ± 12.9 years, and 42.1% were female (Table 1).

**Table 1:** Demographics and baseline characteristics.

Characteristics	*n*	%
Gender		
Male	55	57.9
Female	40	42.1
Age (years)	36.3 ± 12.9	
Ethnic group		
Han	94	98.9
Other	1	1.1
Marital status		
Unmarried	30	31.6
Married	65	68.4
Education level		
Less than high school	9	9.5
High school graduate or general education diploma	16	16.8
College graduate	61	64.2
Any postgraduate	9	9.5
Disease severity (based on self-assessment)		
Mild	28	29.5
Moderate	58	61.1
Severe	9	9.5
Duration of disease (years)		
≤1	23	24.2
1–5	51	53.7
≥5	21	22.1
Comorbidities		
Other gastrointestinal disorders (e.g., chronic gastritis, gastroesophageal reflux disease, etc.)	8	8.4
Other diseases	20	21.1
None	67	70.5

At the end of the 4-week treatment, 68 patients were AR responders, and the other 27 patients were non-responders. A significant difference in the absolute change of the IBS-SSS-C total score before and after treatment was found between the AR responder and non-responder groups (*p* = 0.001). This trend was also shown in 3 items, that is, abdominal pain severity (*p* = 0.032), abdominal pain frequency (*p* = 0.001), and bowel habit satisfaction (*p* = 0.036; Table 2).

**Table 2:** Changes in the IBS-SSS-C total score and each item score before and after treatment in AR responder and non-responder groups.

IBS-SSS-C	AR responder group (*n* = 68)	AR non-responder group (*n* = 27)	*p*
Abdominal pain severity	23.2 (22.7)	11.9 (24.6)	0.032
Abdominal pain frequency	22.4 (33.2)	−1.9 (26.0)	0.001
Abdominal distention severity^*^	19.8 (20.8)	9.2 (20.8)	0.052^†^
Bowel habit satisfaction	13.1 (22.7)	6.9 (22.5)	0.036
Quality of life	16.4 (20.4)	10.1 (21.6)	0.122
Total	90.8 (67.0)	34.2 (82.9)	0.001^†^

^*^Patients without abdominal distension before and after treatment (*n* = 20) were excluded. ^†^Using a student’s *t*-test. AR, adequate relief; IBS-SSS-C, Chinese version of Irritable bowel Syndrome Severity Scoring System.

**Figure 3:**
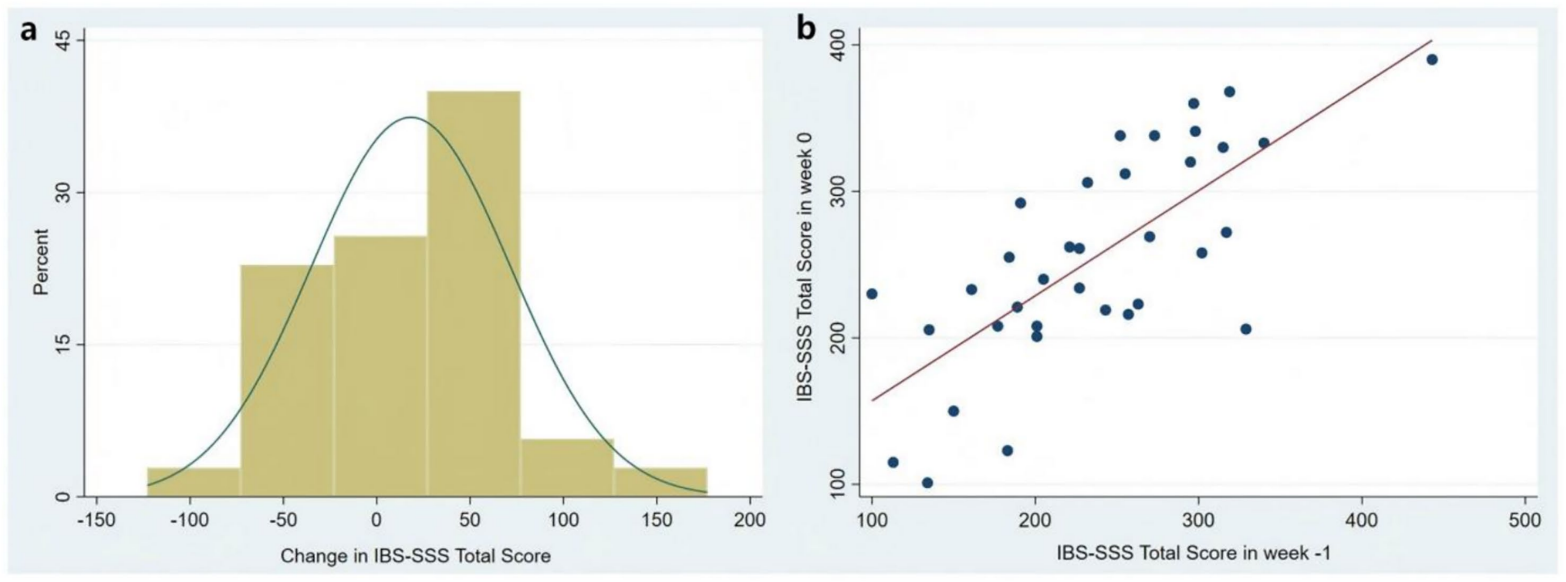
**(a)** Graphical description of the change in IBS-SSS-C total score between two visits (week −1 and week 0). **(b)** The scatterplot corresponding to the correlation between the IBS-SSS-C total score at the two visits (week −1 and week 0). IBS-SSS: Irritable Bowel Syndrome Severity Scoring System.

All 35 patients finished the test–retest reliability assessment with 7–9 days apart. The change in IBS-SSS-C total score between two visits (week −1 and week 0) was 18.3 on average, and the variation range for most patients’ scores was within ± 50. The graphical description of the change in IBS-SSS-C total score between two visits is shown in the Figure 3a; and the scatterplot corresponding to the correlation between the IBS-SSS-C total score at the two visits is shown in the Figure 3b. It shows that the total scores for the two visits were consistent with linear correlation (*p* < 0.001). There was a good correlation (*r* = 0.72) for the IBS-SSS-C total score, and a moderate to good correlation (*r* = 0.41–0.81) for each of the five items (Table 3).

**Table 3:** ICC for the IBS-SSS-C total score and each item score.

IBS-SSS-C	95% confidence interval (CI)	ICC	*p*
Abdominal pain severity	0.10,0.65	0.41	0.006
Abdominal pain frequency	0.30,0.76	0.57	<0.001
Abdominal distention severity	0.31,0.76	0.58	<0.001
Bowel habit satisfaction	0.66,0.90	0.81	<0.001
Quality of life	0.34,0.77	0.60	<0.001
Total	0.50,0.85	0.72	<0.001

ICC, intraclass correlation coefficient; IBS-SSS-C, Chinese version of Irritable Bowel Syndrome Severity Scoring System.

The total IBS-SSS-C score and each item score for all patients at week 0 are shown in the Table 4. There were no floor or ceiling effect in either the IBS-SSS-C or any of its items.

**Table 4:** The floor and ceiling effects of the IBS-SSS-C and each item.

IBS-SSS-C
Mean	SD	Min	Max	25%	50%	75%	% Ceiling effect	% Floor effect
Abdominal pain severity	39.6	19.3	0	100	25	36	51	1.1	2.1
Abdominal pain frequency	48.9	30.2	0	100	20	40	70	13.7	3.2
Abdominal distention severity^*^	35.24	17.3	0	76	25	35	47.5	0	8
Bowel habit satisfaction	59.5	22.2	0	100	35	66	68	3.2	1.1
Quality of life	59.3	21.3	21	100	37	66	68	1.1	0
Total	235.1	73.5	83	443	183	227	295	0	0

^*^Patients without abdominal distension before and after treatment (*n* = 20) were excluded. IBS-SSS-C, Chinese version of Irritable Bowel Syndrome Severity Scoring System.

## 4 Discussion

The IBS-SSS was originally developed and validated in English. In our study, we (1) translated and cross-culturally adapted the Chinese version and (2) validated the IBS-SSS-C for the Chinese population. Our study demonstrated that the IBS-SSS-C had relatively satisfactory responsiveness to change and test–retest reliability. Therefore, the IBS-SSS-C may become a useful tool for IBS symptom severity identification in the Chinese population.

AR has been accepted as an important outcome for IBS global assessment (30–33). We assessed the IBS-SSS-C’s responsiveness to change using AR as an anchor. Our study confirmed that the change in IBS-SSS-C total score before and after treatment was related to symptom relief (i.e., AR). This finding demonstrated that IBS-SSS-C is sensitive to change in symptoms, in accordance with findings in the English- (9), Spanish- (18) and Germany-speaking (17) populations. As for the IBS-SSS-C’s items, the validation results were satisfactory for three items—abdominal pain severity, abdominal pain frequency and bowel habit satisfaction; however, there were no significant differences between AR responder and non-responder groups for the other two items—abdominal distention severity and quality of life. Patients’ misunderstanding of “fu zhang” (“abdominal distension” in Chinese) may explain the insensitivity to change in the abdominal distension severity item. A study has shown that Chinese patients have a poor understanding of “fu zhang” because they might interpret it as postprandial fullness, early satiety, bloating or abdominal distention (34). This could lead to patients’ inaccurate responses on the abdominal distention severity item. Therefore, adequate communication is necessary for clinicians and patients in order to generate an accurate understanding of any items which may be easily confused. The item for quality of life is an investigation of IBS patients’ quality of life. This item has no obvious relationship with symptoms in the short-term, but is related to anxiety and/or depression state. Thus, it is difficult to determine its responsiveness to change over a short time frame. In studies with a long treatment duration or a long follow-up (e.g., 3 months or more), the responsiveness to change for this item may be detectable.

Our results demonstrated generally good test–retest reliability for IBS-SSS-C, similar to the validation results for the versions in three other languages (9, 17, 18). However, the test–retest reliabilities of the abdominal pain severity and abdominal distention severity items were moderate. This may be attributable to the fact that abdominal pain and abdominal distention have an obvious time-varying property. These two symptoms may self-impose remission or aggravation over time. Thus, patients may experience significant changes in their symptoms between visits (1 week ± 2 days). In fact, other language versions of the IBS-SSS have also shown moderate test–retest reliabilities for some items. For example, with regard to the bowel habit satisfaction item, the correlation coefficient for the test–retest reliability of the Spanish version of the IBS-SSS was only 0.45 (18).

A potential limitation of this study was that data were collected only from patients in Guangzhou, China. Which might limit the generalizability of the findings. The single site nature of this study likely limited the external validity of our results. Future multicenter, cross-regional studies with larger samples are needed to further evaluate the applicability and generalizability of the scale.

## 5 Conclusion

IBS-SSS is one of the few validated scales which is widely used to assess IBS symptom severity or IBS global symptoms. We have translated and culturally adapted it into a Chinese version. Our study shows that the IBS-SSS-C appears to be a reliable and appropriate instrument for IBS outcome assessment in the Chinese population. The IBS-SSS-C has been approved by the Rome Foundation. We hope this Chinese version scale will provide practical assistance for clinical studies and routine practice regarding IBS in the Chinese population, though further validation in larger-scale, multi-center studies is needed.

## Data Availability

The original contributions presented in the study are included in the article/Supplementary material, further inquiries can be directed to the corresponding author/s.

## Supplementary material

SUPPLEMENTARY DATASHEET 1 Irritable bowel syndrome severity scoring system (Chinese version).
