# Supplementary material for: Translation, cross-cultural adaptation and validation of the Chinese version of irritable bowel syndrome severity scoring system
Source: Front Med (Lausanne). 2026 May 7;13:1779402. doi: 10.3389/fmed.2026.1779402 (PMC13190209; doi:10.3389/fmed.2026.1779402)
Supplement: SUPPLEMENTARY DATASHEET 1 — Irritable bowel syndrome severity scoring system (Chinese version). [file Data_Sheet_1.PDF]

肠易激综合征问卷

患者姓名：＿＿＿＿＿＿＿＿＿＿＿＿ **主诊医生姓名：**＿＿＿＿＿＿＿＿＿＿

地址：＿＿＿＿＿＿＿＿＿＿＿＿＿＿ **地址：**＿＿＿＿＿＿＿＿＿＿＿＿＿＿

＿＿＿＿＿＿＿＿＿＿＿＿＿＿＿＿＿ ＿＿＿＿＿＿＿＿＿＿＿＿＿＿＿＿＿

联系电话：＿＿＿＿＿＿＿＿＿＿＿＿ **联系电话：**＿＿＿＿＿＿＿＿＿＿＿＿

出生日期：＿＿＿＿＿＿＿＿＿＿＿＿

婚姻状况：未婚/已婚/离异/丧偶/同居

职业：＿＿＿＿＿＿＿＿＿＿＿＿＿＿ **性别：** 男 女

民族：☐汉族 ☐其他 ＿＿＿＿＿＿

父亲职业（含退休）：＿＿＿＿＿＿＿＿＿＿＿＿＿＿＿＿＿＿＿＿＿＿＿＿

填写说明

此表格是为我们记录和监测您的肠易激综合征的严重程度而设计的。考虑到您的症状可能会随时间而发生变化，所以请您尽量根据<u>**目前**</u>（如，最近 10 天左右）的感受回答问题。所有相关资料均会**严格**保密保存。

1. 在有些问题中，有多个不同的回答可能都适合您，请圈出最适合您的哪一个。
2. 有的问题需要您填写贴切的答案。
3. 有些问题需要您在一条直线上划×，以便我们判断该问题的严重程度。

举例：

您的疼痛有多严重？

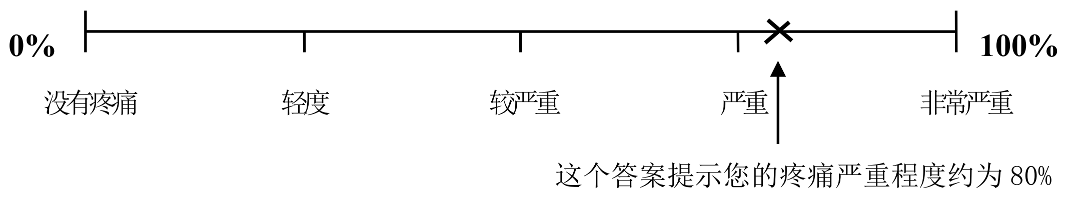

第一部分：严重程度评分

研究人员填写

评分

1. 1）您目前有腹痛（肚子痛）吗？ 有 无 （请圈选适合答案）

2）如果有，您的腹痛（肚子痛）有多严重？

0% ——————————————— 100%

没有疼痛 轻度 较严重 严重 非常严重

3）请填写您在每 10 天中有腹痛的天数。

例如：如果您填写的是 4，表示您每 10 天中有 4 天有腹痛。如果您每天都有腹痛，请填写 10。

有疼痛的天数 ☐ ×10

2. 1）您目前有腹胀*（肚子胀气、膨胀或发紧感）吗？ 有 无 （请圈选适合答案）

（*女性请忽略月经期的腹胀）

2）如果有，您腹部发紧/腹部膨胀的程度有多严重？

0% ——————————————— 100%

没有 轻度 较严重 严重 非常严重

3. 您对自己排便习惯的满意度是多少？

0% ——————————————— 100%

非常满意 较满意 不满意 非常不满意

4. 您觉得您的肠易激综合征在多大程度上影响或干扰了您的日常生活？

0% ——————————————— 100%

没有影响 有点影响 明显影响 完全影响

肠易激综合征症状严重度评分： ☐

第二部分：肠易激综合征的其他资料

排便习惯

5. 1） **您每天/每周/每月排便次数最多是多少次？**

排便次数 ☐ 每天/每周/每月（请圈选适合的时间单位）

注意：对部分患者问题 1)和问题不 2)的答案可能是相同的

2） **您每天/每周/每月排便次数最少是多少次?**

排便次数 ☐ 每天/每周/每月（请圈选适合的时间单位）

6. 下列问题您可圈选 1 个以上的答案：

您的大便性状是：

1）**正常**	经常/偶尔/从不（请圈选适合的答案）
2）**干硬**	经常/偶尔/从不（请圈选适合的答案）
3）**很细（线状）**	经常/偶尔/从不（请圈选适合的答案）
4）**小块状（像兔子的球状粪便）**	经常/偶尔/从不（请圈选适合的答案）
5）**糊状（粥样）**	经常/偶尔/从不（请圈选适合的答案）
6）**水样**	经常/偶尔/从不（请圈选适合的答案）

7. 下列问题您可圈选 1 个以上的答案：

**您是否曾经：**	请圈选适合的答案	
1）**大便带黏液（或黏胨，或果酱样物）**	是	否
2）**大便带血**	是	否
3）**排便急迫感**	是	否
4）**排便费力**	是	否
5）**排便后仍有不尽感**	是	否

疼痛部位

请根据您的疼痛的部位在下图中的对应位置画“×”
（需要时，可在下图多个部位画“×”）

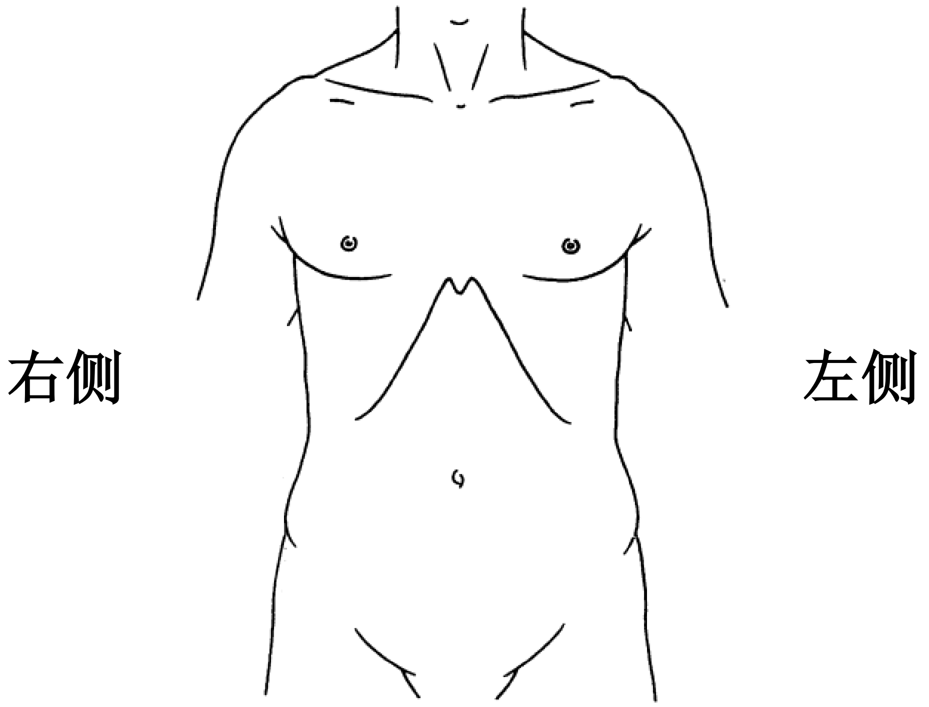

8. 您是否曾经：

1）注意到当您有腹痛的时候，排便更频繁或者大便变稀 是 否

请圈选适合的答案

2）注意到腹痛是否通常在排便后改善 是 否

请圈选适合的答案

9. 在过去的 1 年时间里，您大约有几个星期：

注意：以自然星期算，只要这个星期有症状，就计算为 1 个星期。

1） 由于肠易激综合征而不能上班 ____________

（如果您因为肠易激综合征而完全放弃了工作，请填写 52）

2） 工作中受肠易激综合征的困扰 ____________
